# Regulatory T cells as a biomarker for response to adalimumab in rheumatoid arthritis

**DOI:** 10.1016/j.jaci.2018.04.026

**Published:** 2018-09

**Authors:** Dao X. Nguyen, Alice Cotton, Laura Attipoe, Coziana Ciurtin, Caroline J. Doré, Michael R. Ehrenstein

**Affiliations:** a Division of Medicine, Centre for Rheumatology, University College London, London, United Kingdom; b Comprehensive Clinical Trials Unit at UCL, Institute of Clinical Trials and Methodology, London, United Kingdom

To the Editor:

Despite the success of anti-TNF therapy for patients with rheumatoid arthritis (RA), around one-third of patients fail to benefit from this treatment.^1^ There is an intensive search for biomarkers, mostly on an empirical basis, that will guide the use of anti-TNF therapy to those patients with RA most likely to respond.^2^ Ineffective treatments allow inflammation to persist, resulting in joint damage and disability. A method to predict response to anti-TNF therapy would be cost-effective and minimize delays in receiving an efficacious treatment, therefore representing a step toward precision medicine for patients with RA.

We have previously shown that CD4 regulatory T (Treg)-cell numbers and suppressor function were enhanced in the peripheral blood of patients with RA who responded to anti-TNF antibody therapy (adalimumab) in contrast to patients responding to the soluble TNF receptor etanercept.^3^ We developed an *in vitro* assay that led to the hypothesis that Treg-cell-monocyte interactions via TNF-TNFRII were pivotal to the immunomodulatory actions of anti-TNF antibody blockade in RA.^4^ We recruited a cohort of patients with RA about to commence treatment with adalimumab (see Table E1 in this article's Online Repository at www.jacionline.org) to determine whether this TNF inhibitor's ability to boost Treg cells *in vitro* using PBMCs before treatment would predict clinical response.

We first sought to identify any significant correlation between the anti-TNF antibody-induced Treg-cell changes *in vivo*^3^ after therapy with adalimumab and *in vitro*^4^ before therapy. The change in the percentage of CD4 Treg cells *in vitro* at baseline correlated with the shift in the frequency of circulating Treg cells in those patients at 3 months after anti-TNF antibody therapy (see Fig E1, *A*, in this article's Online Repository at www.jacionline.org).

We next tested whether this *in vitro* Treg-cell assay could predict clinical response. Adalimumab boosted the proportion (Fig 1, *A*) and absolute number (Fig E1, *B*) of CD4 Treg cells by at least 40% *in vitro* in 12 of the 14 patients who went on to respond to this biologic therapy at 3 months but not in any of the patients who did not respond to this treatment. Those patients who responded continued to do so at 6 and 12 months although only the 3-month data set was complete because some of the patients had an intercurrent infection at 6 and 12 months and had to temporarily stop the adalimumab. All the nonresponders had stopped adalimumab by 6 months. There was a significant difference between responders and nonresponders (*P* = .016) with respect to the changes in Treg cells following *in vitro* stimulation with adalimumab (see Table E2 in this article's Online Repository at www.jacionline.org). Logistic regression analysis to assess predictive power with respect to clinical response yielded high sensitivity and specificity (area under the curve [AUC], 0.87) for the shift in CD4 Treg-cell frequency in the baseline PBMC sample stimulated by adalimumab *in vitro* (Fig 1, *B*; see Table E3 in this article's Online Repository at www.jacionline.org). We wondered whether the 2 patients who responded to adalimumab without a rise in their Treg-cell frequency *in vitro* did so because of the lack of combination treatment with methotrexate, which has been shown to have Treg-cell immunomodulatory properties.^5^ Once the cohort of responders was stratified according to the use of concomitant methotrexate therapy, both patients who responded without increasing their Treg-cell frequency *in vitro* were treated with adalimumab monotherapy (Fig E1, *C*). Patients who had greater than a 40% rise in CD4 Treg cells showed a significant reduction in C-reactive protein after 3 and 6 months of therapy (Fig 1, *C*).

**Fig 1:**
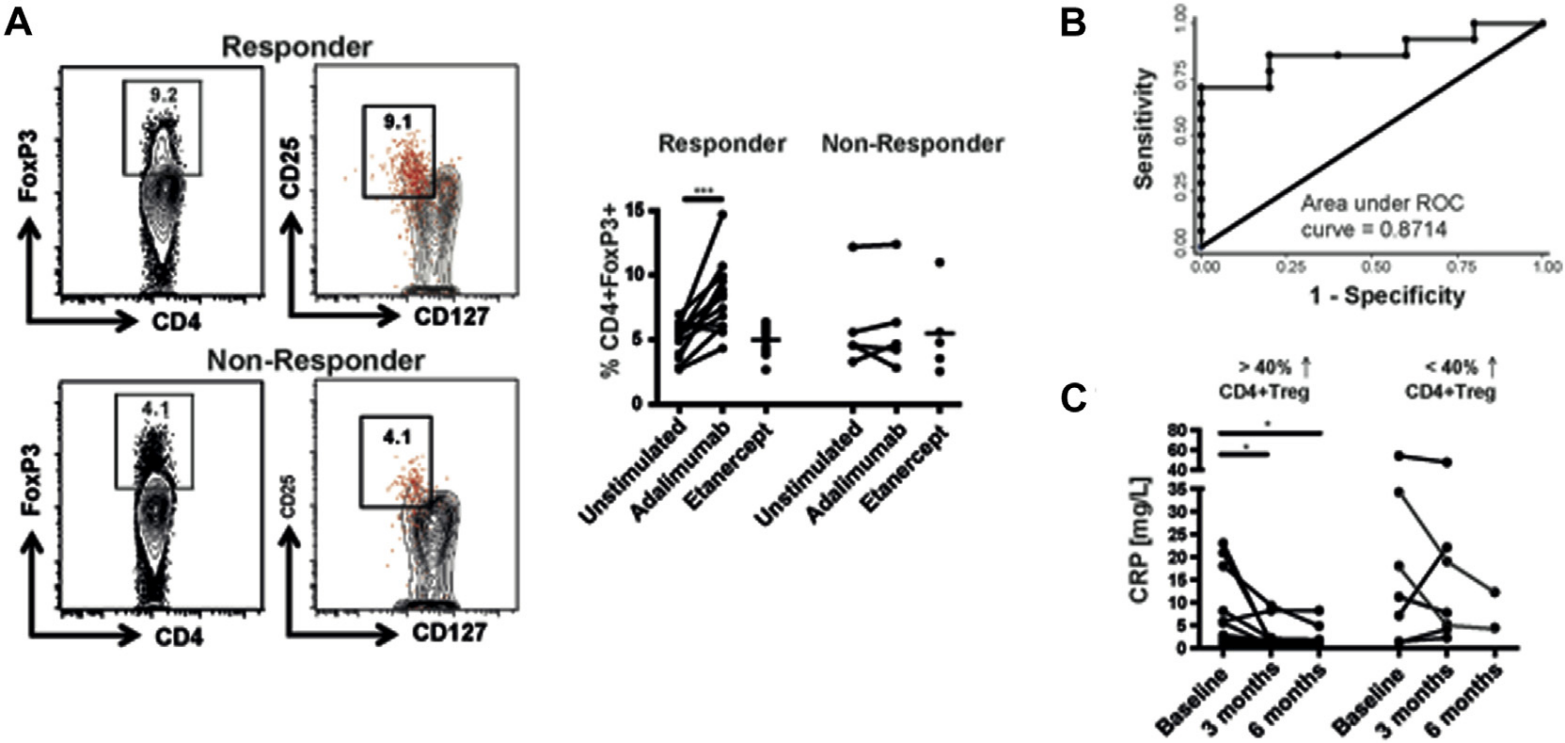
Adalimumab-driven increase in CD4 Treg cells in PBMCs from patients with RA *in vitro* predicted subsequent clinical response to therapy. **A,** Representative FACS plot indicating the percentage of CD4^+^Foxp3^+^ Treg cells in PBMCs stimulated with adalimumab *in vitro* from a patient who subsequently responded and a patient who did not respond to adalimumab therapy assessed at 3 months. The right-hand panel shows that Foxp3^+^ CD4 T cells cosegregate with CD127^lo^CD25^hi^ CD4 T cells. The corresponding cumulative data of Treg-cell frequency in PBMCs from patients who responded (n = 14) or not (n = 5) to adalimumab therapy cultured with adalimumab or etanercept. **B,** Receiver-operating characteristic (ROC)-curve analysis of the percentage increase in Treg cells predicting clinical response (n = 19). **C,** Serum CRP before and after therapy in patients divided according to whether adalimumab increased Treg-cell frequency by more than 40% in the baseline sample *in vitro* (n = 19). CRP values for 2 responding patients who temporarily stopped their adalimumab at 6 months because of infection come from data collected between 6 and 9 months. *CRP*, C-reactive protein. *P < .05, ***P < .001 by paired *t* test.

We hypothesized that elevated expression of baseline monocyte membrane TNF, to which adalimumab binds,^4^ would be associated with increased Treg-cell frequency *in vitro* and predict clinical response. Indeed, there was a significant correlation between the pretreatment monocyte membrane TNF expression and the change in the percentage of CD4 Treg cells stimulated by adalimumab *in vitro* (see Fig E2, *A*, in this article's Online Repository at www.jacionline.org). The baseline expression of monocyte membrane TNF in PBMCs was significantly higher in patients responding to adalimumab compared with those whose disease activity did not improve (Fig E2, *B*). Stratifying the responding patients according to concurrent treatment with methotrexate revealed that monocyte membrane TNF expression was elevated at baseline in those patients receiving methotrexate compared with those not on this treatment (Fig E2, *C*). Moreover, methotrexate directly enhanced membrane TNF expression *in vitro* in PBMCs from patients with RA before treatment with this conventional disease-modifying antirheumatic drug (Fig E2, *D*).

Logistic regression analysis to assess the predictive power of baseline membrane TNF expression with respect to clinical response yielded high sensitivity and specificity (AUC, 0.87) (Fig E2, *E*; see Table E3). These results translated into differential binding of adalimumab to monocytes in the baseline sample between subsequent responders and nonresponders (Fig E2, *F*). Adalimumab binding was also strongly predictive of clinical response (AUC, 0.91) (Fig E2, *G*; Table E3). Adalimumab not only bound to membrane TNF but also increased its expression after 3 days in culture in responding patients (Fig E2, *H*). There were no significant differences between the AUC for the 3 prediction models (increase in Treg cells, monocyte membrane TNF expression, and adalimumab binding).

We next sought to elucidate the underlying signaling mechanism triggered within monocytes by adalimumab. We investigated the role of p38 signaling in modulating monocyte membrane TNF expression by adalimumab because of its role in reverse signaling via membrane TNF.^6^ *In vitro*, adalimumab, but not etanercept, enhanced p38 MAP kinase phosphorylation in monocytes from patients treated with methotrexate, which was associated with an increase in Treg cells (see Fig E3, *A*, in this article's Online Repository at www.jacionline.org). Mirroring the *in vitro* results, monocytes from patients responding to adalimumab therapy displayed enhanced p38 phosphorylation (Fig E3, *B*) compared with patients treated with methotrexate. Adalimumab did not alter total p38 expression either *in vitro* (see Fig E4, *C*, in this article's Online Repository at www.jacionline.org) or *in vivo* (Fig E4, *D*). The expression of membrane TNF correlated with p38 phosphorylation in monocytes stimulated by adalimumab (Fig E3, *C*). There was also a significant correlation between monocyte membrane TNF and p38 phosphorylation *in vivo* in patients responding to adalimumab treatment but not in patients responding to methotrexate (Fig E3, *D*).

As p38 MAP Kinase can regulate IL-10 production in monocytes,^7^ we examined whether adalimumab triggered the production of this immunoregulatory cytokine by monocytes. Adalimumab, but not etanercept, stimulated IL-10 production by monocytes *in vitro*, which was dependent on p38 phosphorylation (Fig E3, *E*). Similarly, IL-10 production from monocytes was increased *in vivo* in patients responding to adalimumab therapy (Fig E3, *F*). Blockade of p38 phosphorylation or IL-10 abrogated the adalimumab-driven increase in membrane TNF (Fig E3, *G*) and the associated rise in Treg-cell frequency (Fig E3, *H*). Very little IL-10 production was detected by CD4 Treg cells following stimulation with adalimumab (data not shown).

Our findings highlight the value in understanding the mechanism of action of biologic therapies, which facilitates the rational development of clinical response biomarkers.^8^ These results indicate that monocyte membrane TNF expression at baseline determines a patient's response to adalimumab therapy, which may be applicable to other therapeutic anti-TNF antibodies. Monocyte membrane TNF expression, its binding to adalimumab, which in turn increased the CD4 Treg-cell frequency *in vitro*, all predicted the subsequent response of patients with RA to adalimumab. The almost identical AUC for these 3 biomarkers suggests that they are likely modulated along the same pathway by adalimumab to improve clinical outcome. The increase in Treg cells was more consistently associated with a clinical response if patients were also treated with methotrexate, which is well established to have a synergistic benefit with adalimumab.^9^ Indeed, methotrexate directly enhanced monocyte membrane TNF expression, thereby priming patients to respond to adalimumab. Further investigations are required to confirm these results in larger cohorts but raise the prospect of developing a biomarker, or set of biomarkers, which would direct therapy to those most likely to respond, thereby aligning with the principles of personalized medicine. Moreover, these data validate Treg cells as a therapeutic target in RA, and identify novel therapeutic approaches, such as directly boosting membrane TNF expression.

## Figures and Tables

**Fig E1:**
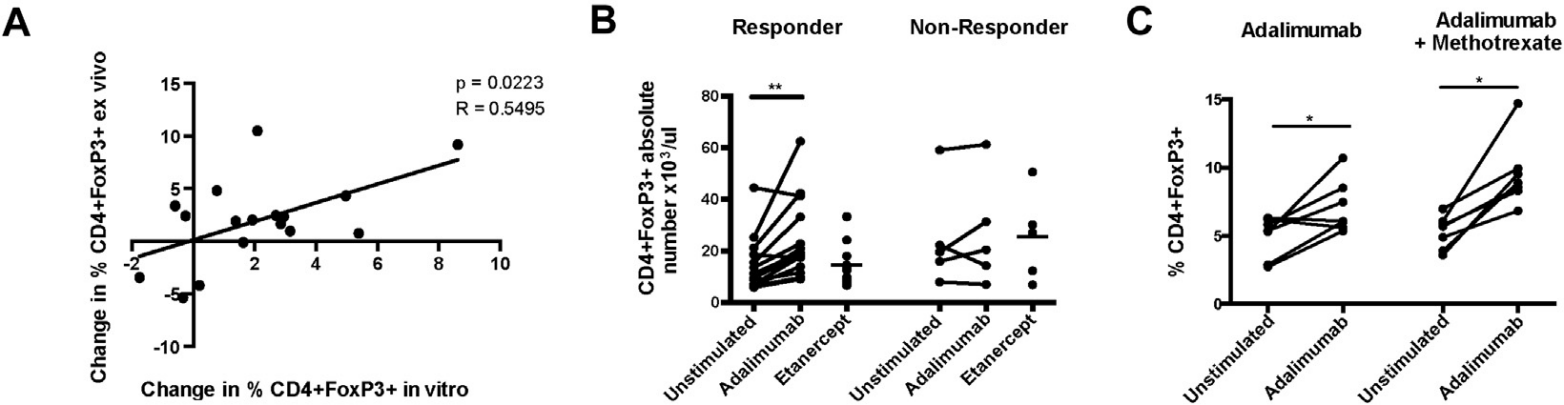
**A,** Correlation between the change in CD4 Treg-cell frequency (proportion of CD4^+^ T cells) in PBMCs from patients at baseline cultured with adalimumab *in vitro* and the change in frequency of peripheral blood Treg cells in the same patients after 3 months of adalimumab therapy. **B,** Absolute number of Treg cells on day 3 from PBMCs from patients who responded (n = 14) or not to adalimumab therapy assessed at 3 months (n = 5) cultured with either anti-TNF agent. **C,** Percentage of Treg cells in PBMCs at baseline from patients who responded to therapy (n = 14) divided according to whether they were treated with methotrexate in combination with adalimumab. *P < .05, **P < .01 by paired *t* test.

**Fig E2:**
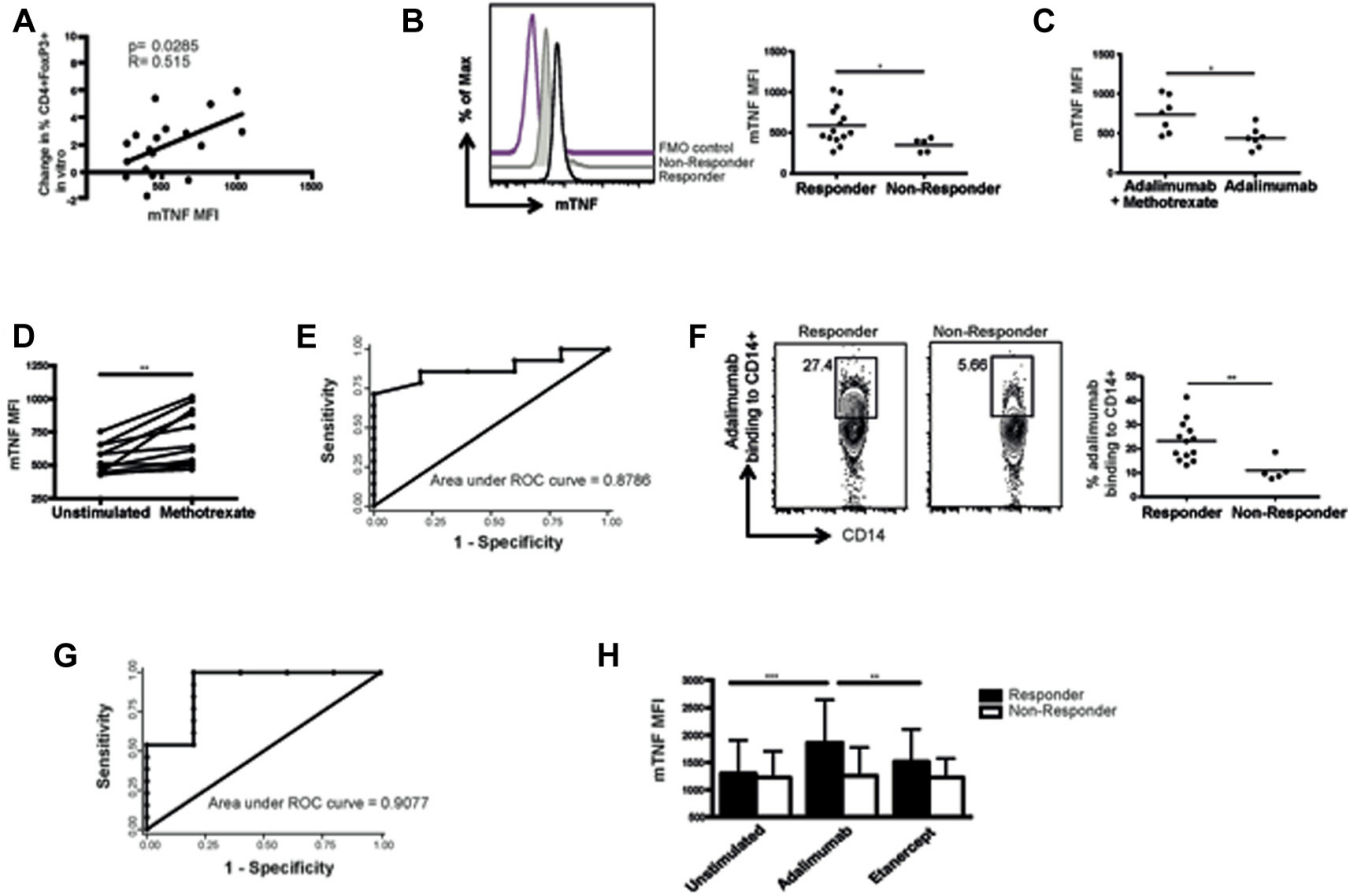
Increased monocyte membrane TNF expression and adalimumab binding predicted therapeutic response. **A**, Correlation between the change in the percentage of Treg *in vitro* stimulated by adalimumab and baseline monocyte membrane TNF expression in patients before adalimumab treatment (n = 19). **B,** Membrane TNF expression on CD14^+^ monocytes isolated before adalimumab treatment from responders (n = 14) and nonresponders to therapy (n = 5). **C,** Monocyte membrane TNF expression at baseline from patients who responded to adalimumab therapy (n = 14), divided according to concurrent methotrexate therapy. **D,***In vitro* effect of methotrexate on monocyte membrane TNF expression in PBMCs from untreated patients with RA (n = 12). **E,** ROC-curve analysis of the utility of baseline membrane TNF expression before therapy to predict response to adalimumab (n = 19). **F,** Adalimumab binding to monocytes from patients before treatment divided according to their clinical response to adalimumab (n = 18). **G,** ROC-curve analysis of the percentage binding of adalimumab to monocytes to predict response to adalimumab (n = 18). **H,** Monocyte membrane expression after culture with adalimumab or etanercept using PBMCs taken before adalimumab therapy, divided according to subsequent response (n = 14) or not (n = 5) to adalimumab. *ROC*, Receiver-operating characteristic. *P < .05; **P < .01; ***P < .001 by paired *t* test or Mann-Whitney test.

**Fig E3:**
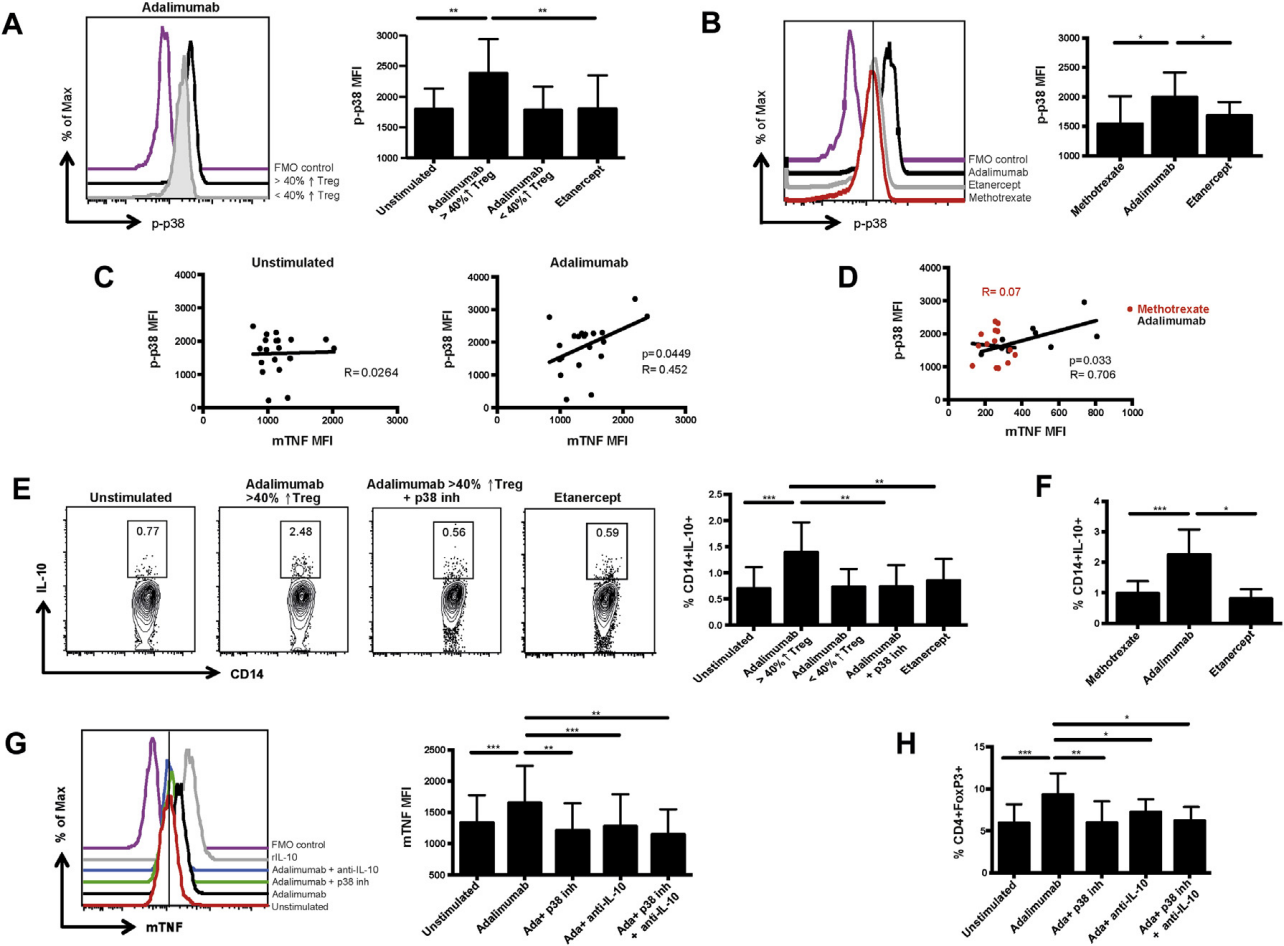
Adalimumab enhanced monocyte membrane TNF expression and increased Treg-cell frequency through an IL-10/p38-dependent mechanism. **A,** Monocyte p-p38 expression in methotrexate-treated RA PBMCs after stimulation with adalimumab *in vitro*. The results were divided according to whether the frequency of CD4^+^Foxp3^+^ Treg cells increased by more than 40% (n = 14) or not (n = 5) in response to adalimumab. **B,***Ex vivo* p-p38 in CD14^+^ monocytes from patients responding to methotrexate (n = 14), adalimumab (n = 9), or etanercept (n = 5). **C,** Correlation between monocyte membrane TNF and p-p38 expression in PBMCs from patients treated with methotrexate cultured with adalimumab (n = 20). **D,***Ex vivo* correlation between monocyte membrane TNF and p-p38 expression in monocytes from patients responding to methotrexate (n = 14) or to adalimumab therapy (n = 9). **E,** Representative FACS plot and cumulative data of IL-10 production by monocytes stimulated with adalimumab ± a p38 inhibitor (inh), or stimulated with etanercept (n = 9). **F,***Ex vivo* IL-10 production by monocytes from patients responding to DMARD (n = 15), to adalimumab (n = 7), or to etanercept (n = 3). The effects of p38 inhibition and/or IL-10 blockade on **(G)** monocyte membrane TNF expression and **(H)** Treg-cell frequency from patients who responded to adalimumab (n = 7). *DMARD*, Disease-modifying antirheumatic drug. *P < .05; **P < .01; ***P < .001 by paired *t* test or Mann-Whitney test.

**Fig E4:**
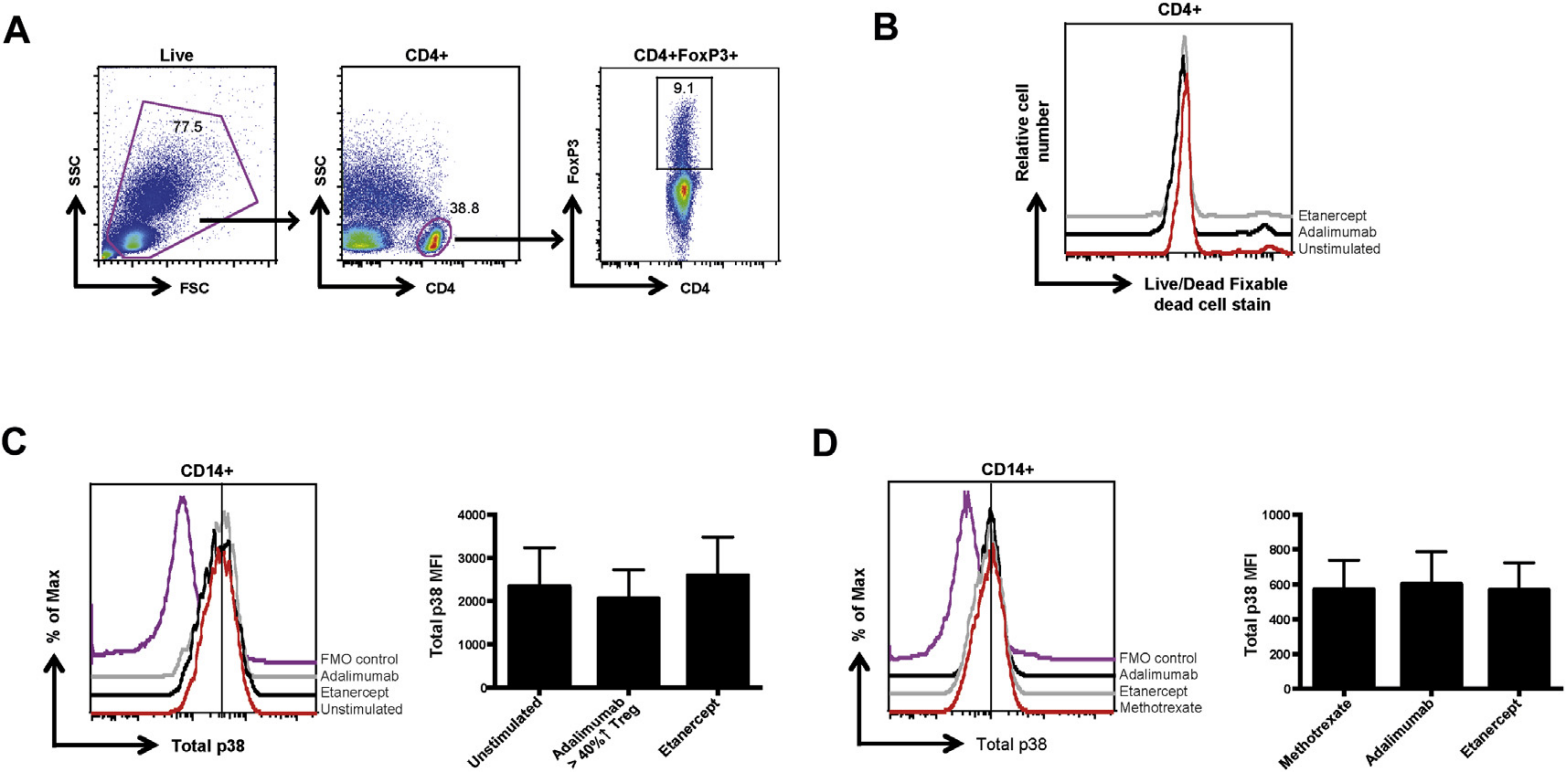
Flow cytometry gating strategy for CD4^+^FoxP3^+^ Treg cells and total p38 expression within monocytes. **A,** Gating strategy to reveal CD4^+^Foxp3^+^ Treg cells following *in vitro* stimulation with adalimumab. **B,** Representative histograms of CD4^+^ T cells stained with LIVE/DEAD fixable dead cell stain, from an RA PBMC sample cultured with adalimumab, etanercept, or without stimulation for 3 days. **C,** Histogram and cumulative data of total p38 expression in CD14^+^ monocytes in PBMCs from patients with RA treated with methotrexate following stimulation with adalimumab, where Treg cells increased by more than 40% or etanercept *in vitro* (n = 8). **D,** Total p38 expression from PBMCs isolated *ex vivo* from different patient groups (methotrexate, n = 14; adalimumab, n = 6; etanercept, n = 5). *FSC*, Forward scatter; *SSC*, side scatter.

**Table E1:** Patient with RA disease activity and treatment from the longitudinal study

Responder (R) or nonresponder (NR)	Concomitant treatment	DAS28 baseline	DAS28 3 mo
R	Nil	5.64	3.89
R	Nil	6.08	2.11
R	Nil	4.13	2.55
R	Nil	7.2	4.52
R	Nil	5.17	1.63
R	Nil	5.18	2.62
R	LEF, HCQ	5.18	3.67
R	MTX	5.32	2.56
R	MTX	5.27	2.14
R	MTX, SSZ	5.92	3
R	MTX, Pred	7.15	4.12
R	MTX, SSZ	5.61	3.27
R	MTX, HCQ	5.37	3.98
R	MTX, HCQ, SSZ	6.45	5.19
NR	Nil	5.17	5.75
NR	LEF	5.62	4.89
NR	Pred, SSZ	6	5.19
NR	MTX	5.1	4.36
NR	MTX, Pred	5.16	5.83

Nil indicates that the patient was not taking any disease-modifying antirheumatic drug.

DAS28 = 0.56 × √(tender28) + 0.28 × √ (swollen28) + 0.70 × ln(CRP) + 0.014 × VAS_patient_. See https://www.das-score.nl/das28/en/introduction-menu.html. All patients who were taking MTX were on a constant dose for the last 5 months before the sample was taken.

*CRP*, C-reactive protein; *HCQ*, hydroxychloroquine; *LEF*, leflunomide; *MTX*, methotrexate; *Pred*, prednisolone; *SSZ*, sulphasalazine.

**Table E2:** *t* tests comparing changes in CD4 Treg-cell and monocyte membrane TNF in response to anti-TNF stimulation

Variable	Anti-TNF	Anti-TNF—unstimulated								
		Nonresponders (N = 5)			Responders (N = 14)			Responders – nonresponders		
		Difference	95% CI	*P* value	Difference	95% CI	*P* value	Difference	95% CI	*P* value
Treg cell	Adalimumab	0.1	−1.4 to 1.5	.92	**3.1**	**1.7 to 4.5**	**.0003**	**3.1**	**0.7 to 5.5**	**.016**
Treg cell	Etanercept	−0.5	−1.3 to 0.3	.13	−0.1	−0.9 to 0.6	.68	0.4	−0.9 to 1.7	.52
mTNF	Adalimumab	35	−198 to 268	.69	**303**	**114 to 492**	**.004**	267	−62 to 597	.11
mTNF	Etanercept	−0.4	−360 to 364	.99	95	−126 to 235	.37	95	−304 to 494	.62

Paired *t* tests were performed for each anti-TNF agent to compare stimulated with anti-TNF and unstimulated values for responders and nonresponders separately. Two sample *t* tests were used to compare the anti-TNF agent stimulated-unstimulated differences in responders and nonresponders. *P* values <.05 are highlighted in bold.

*mTNF*, MembraneTNF.

**Table E3:** Logistic regression predicting clinical response to therapy

Variable	Predictor	N	AUC	AUC 95% CI	Cutoff	% Sensitivity	% Specificity
**Treg cell**	**Adalimumab: unstimulated**	**19**	**0.87**	**0.70-1.00**	**40% increase**	**71.4**	**80.0**
Treg cell	Etanercept: unstimulated	19	0.56	0.27-0.84	**40% increase**	14.3	100.0
mTNF cell	Adalimumab: unstimulated	19	0.69	0.34-1.00	**40% increase**	21.4	100.0
mTNF cell	Etanercept: unstimulated	19	0.61	0.26-0.97	**40% increase**	14.3	80.0
**mTNF cell**	***Ex vivo* expression**	**19**	**0.87**	**0.70-1.00**	**420**	**78.6**	**80.0**
**Adalimumab**	***Ex vivo* binding**	**18**	**0.91**	**0.71-1.00**	**14**	**92.3**	**80.0**

Logistic regression analysis was performed to predict response (yes/no). A number of alternative models were investigated. The receiver-operating characteristic (ROC) curve for each model plots sensitivity against 1 − specificity using each observed value of the predictor to define a positive test result. Summary measures are the AUC and its 95% CI. The AUC has a maximum value of 1 and a minimum value of 0; an AUC of 0.5 would indicate a predictor with no discriminating ability. For percentage change variables, a greater than 40% increase was used to define a positive test result. For each cutoff, the corresponding values of the sensitivity and specificity are presented. The 3 models with the highest values for the AUC have been highlighted in bold. For data using adalimumab in the *in vitro* assay, a cutoff of a 40% increase was used to define a positive test result when calculating the sensitivity and specificity presented in the table. For *ex vivo* analysis, the table presents the sensitivity and cutoff corresponding to a specificity of 80%.

*mTNF*, MembraneTNF.

## Methods

### Patient populations

Nineteen patients with active RA whose diagnosis fulfilled the American College of Rheumatology 1987 revised classification criteria for RA^E1^ who were about to commence adalimumab therapy and had a Disease Activity Score (DAS28-CRP)^E2^ of 5.1 or more were recruited. Clinical details of these patients are provided in Table E1. Clinical response at 3 months of treatment was defined as a decrease of more than 1.2 in DAS28 according to the EULAR response criteria.^E3^ We also recruited a cross-sectional cohort of patients with RA on conventional disease-modifying antirheumatic drugs including methotrexate, adalimumab, or etanercept. Informed consent was taken from all patients. This study was approved by the National Research Ethics Committee (Reference 13/LO/0999).

### Antibodies and reagents

The following antibodies/reagents were used: Alexa Fluor 700–conjugated CD4 (RPA-TA, BD Biosciences, Franklin Lakes, NJ), Pacific blue–conjugated CD14 (M5E2, BD Biosciences), allophycocyanin (APC)-Cy7–conjugated CD25 (M-A251, BD Biosciences), phycoerythrin (PE)-Cy5–conjugated CD127 (eBioRDR5, eBioscience, San Diego, Calif), PE-conjugated FoxP3 (236A/E, eBioscience), fluorescein isothiocyanate (FITC)-conjugated membrane TNF (FAB210F, R&D Systems, Minneapolis, Minn), PE-Cy7–conjugated p-P38 (36/p38, BD Biosciences), PE-conjugated total P38 (D13E1, New England Biolabs, Ipswich, Mass), PE-conjugated IL-10 (JES3-9D7, eBioscience), anti–IL-10 (25209, R&D Systems), P38 MAP kinase inhibitor (SB 202190, Sigma-Aldrich, St Louis, Mo), and LIVE/DEAD Fixable Blue dead cell stain (Invitrogen, Carlsbad, Calif).

### Stimulation with anti-TNF agents and methotrexate

Adalimumab or etanercept was added at a final concentration of 10 μg/mL and methotrexate was added at a concentration of 50 μg/mL to PBMCs for 3 days. Anti-CD3 stimulation was not used. Fig E4, *A*, shows the gating strategy to detect CD4^+^Foxp3^+^ Treg cells and a representative histogram shows the LIVE/DEAD Fixable dead cell stain following *in vitro* stimulation with adalimumab compared with etanercept (Fig E4, *B*). Most Foxp3^+^ Treg cells (red) cosegregate with CD127^lo^CD25^hi^ CD4 T cells (Fig 1, *A*).

### Flow cytometric analysis

Cell surface and intracellular staining were performed on PBMCs in accordance with manufacturers’ instructions. The eBioscience FoxP3 staining buffer set was used for all intracellular staining. The production of IL-10 by monocytes gated on CD14^+^ monocytes was determined by flow cytometry after 4 hours of stimulation with 4 μg/mL phorbol myristate acetate, 1 μg/mL ionomycin, and 2 μg/mL Golgi Stop (BD Biosciences) both *in vitro* using PBMCs following stimulation with adalimumab and *ex vivo* analysis of freshly isolated PBMCs.

### P38 MAP Kinase PhosFlow

PBMCs were fixed with equal volume of warm BD Phosflow Fix buffer I for 10 minutes at 37°C and then permeabilized for 10 minutes by cold BD Phosflow Perm/wash buffer III at 4°C, followed by surface CD4, CD14 and intracellular Foxp3, and p-p38 staining. *Ex vivo* analysis was performed within 3 hours of venepuncture.

### Biotinylation binding assay

Biotinylation of adalimumab was performed in accordance with Thermo Scientific's (Waltham, Mass) instructions of the EZ-Link NHS-PEO Solid-phase Biotinylation kit. Biotinylated adalimumab was added to PBMCs from patients with RA for 30 minutes before staining for CD14. Cells were then resuspended in PE-conjugated streptavidin for 30 minutes before acquisition by FACS.

### Statistical analysis

Groups of continuous variables were compared with paired *t* tests, Mann-Whitney tests, or unpaired *t* tests using GraphPad (La Jolla, Calif) Prism software (Table E2). Geometric means were used to calculate significance for mean fluorescence intensity (MFI) values generated by Flow Cytometry. Logistic regression analysis to predict clinical response was carried out using Stata Release 14 (StataCorp, College Station, Tex). The models investigated are summarized in Table E3. *P* values of less than .05 were considered statistically significant. Associations between continuous variables were summarized with Pearson correlation coefficients. *P* values are denoted in figures as follows: **P* < .05, ***P* < .01, and ****P* < .001.

## Discussion

There are few data on the distinct immune effects of etanercept and adalimumab, or the other anti-TNF antibodies, in the context of RA. Indeed given their equivalent efficacy in RA, there has been little impetus to explore any potential differences. In stark contrast, etanercept inefficacy in Crohn disease, as opposed to adalimumab and the other anti-TNF antibodies, has spurred investigators to rationalize this unexpected outcome; the differential binding to membrane TNF has been suggested as a possible explanation.^E4^ Consistent with these observations, we have shown that adalimumab but not etanercept binds to membrane TNF expressed by monocytes from patients with RA.^E5^ Monocytes from patients with RA are known to have elevated monocyte membrane TNF expression compared with healthy controls, and anti-TNF antibodies modulate cytokine production by monocytes from patients with RA but not healthy controls.^E6^ Our findings reported here indicate that the level of expression of membrane TNF can predict whether a patient will respond to adalimumab therapy. These results raise the possibility that adalimumab's effects on membrane TNF expressed by monocytes underlie its mechanism of action in Crohn disease and in RA.

Anti-TNF antibody therapy is known to enhance p38 phosphorylation in cell lines engineered to express membrane TNF^E7^ and in colonic mucosa taken from patients with Crohn disease.^E8^ Furthermore, p38 phosphorylation in the colonic mucosa distinguished between patients with Crohn disease who responded and those who did not respond to infliximab.^E9^ We noted a significant correlation between p38 phosphorylation and membrane TNF expression only in patients with RA responding to adalimumab. A study to examine whether genetic polymorphisms related to p38 signaling contributed to the variable response of TNF antagonists in RA revealed greater association of p38-associated single nucleotide polymorphisms with response to adalimumab compared with etanercept.^E10^ In agreement with our results, the ligation of membrane TNF has been reported to mediate up to a 6-fold increase in TNF-mRNA expression and activation of p38 in monocytes.^E8^ Reverse signaling through membrane TNF by anti-TNF antibodies not only activated p38 phosphorylation but also IL-10 production consistent with previous studies using a T-cell line–expressing membrane TNF.^E11^ Indeed, monocyte p38 phosphorylation stimulates the production of various proinflammatory and anti-inflammatory cytokines, including IL-10.^E12^ Our data support the notion that membrane TNF is an example of a cytokine with immunoregulatory properties that is modulated by a p38/IL-10 signaling pathway. Intriguingly, IL-10 has been reported to specifically increase membrane TNF expression without affecting soluble TNF production,^E13^ suggesting that IL-10 and membrane TNF may act synergistically to suppress immune responses.

Adalimumab had no Treg-cell–enhancing effect on healthy PBMCs,^E5^ drawing parallels with the actions of IL-10 in a murine colitis model that boosted Treg cells, and Foxp3 expression, only in the presence of inflammation.^E14^ Myeloid cells were the predominant source of IL-10 in this murine model of colitis. The precise effects of inflammation were not investigated in this colitis study, leaving open the possibility that membrane TNF expressed by myeloid cells could play a pivotal role in driving Treg-cell expansion. The blockade of either IL-10 or p38 phosphorylation in our studies prevented the increase in monocyte membrane TNF expression and Treg-cell frequency in RA, suggesting a causal role for this immunoregulatory pathway. In summary, we propose a positive feedback loop whereby adalimuamb stimulates further increases in monocyte membrane TNF which is dependent on p38 phosphorylation and IL-10. This elevation in monocyte membrane TNF expression mediates the response to anti-TNF antibody in RA through boosting Treg-cell numbers and function.
